# Post-Modern Epidemiology: Back to the Populations

**DOI:** 10.3390/epidemiologia1010002

**Published:** 2020-08-19

**Authors:** Arnaud Chiolero

**Affiliations:** 1 Population Health Laboratory (#PopHealthLab), University of Fribourg, Route des Arsenaux 41, 1700 Fribourg, Switzerland; arnaud.chiolero@unifr.ch; 2 Institute of Primary Health Care (BIHAM), University of Bern, 3012 Bern, Switzerland; 3 Department of Epidemiology, Biostatistics, and Occupational Health, McGill University, Montreal, QC H3A 1A2, Canada

The creation of new journal about epidemiology is a good opportunity to think about the state of the field and to make proposals for its development. I argue here that it is time for post-modern epidemiology.

Epidemiology has moved from “traditional” to what is called “modern epidemiology” with the development of risk factor epidemiology [1] (Table 1). The risk factor approach was key, notably, to identify major and modifiable causes of cardiovascular diseases and to help prevent them. It was also aligned with the motivation for epidemiologists to engage in science, *explanation* being at the heart of modern epidemiology. This approach failed, however, to address the social, economic, and environmental determinants of health. Further, it moved the field of epidemiology from a population perspective to an individual one [1].

Other trends with a major impact on epidemiology are the development of data–science and the growing access to big and highly heterogenous health-related data [2]. These data are changing observational research as well as the field of public health surveillance and population health monitoring through a widening scope of applications and new methods. Despite an unprecedented access to data, the Covid-19 pandemic has, however, revealed the failure of most public health surveillance systems worldwide, making their reinventions necessary.

Further, while there is a renewed interest in social and environmental epidemiology, notably through the development of life course epidemiology [3], the defiance toward epidemiology has never been so strong [4] and this is due to the major failures of some observational studies, the enduring confusion between association and causality, the large number of studies reporting false-positive results [5], and the production of low value research without public health relevance [6,7].

Within this context, I would like to make three proposals for the development of post-modern epidemiology.

Firstly, we should re-enchant public health surveillance and population health monitoring and put *description* at the forefront of epidemiology. Opportunities through the developments of data science are huge; new data and new methods for visualization or mining, as well as machine learning and artificial intelligence, are transforming the field of epidemiology and surveillance [2]. These new and “big” data, however, do not speak by themselves more than “small” data, and making sound epidemiological research and providing information useful for the decision in public health requires, more than ever, a training that goes far beyond the mastery of data management and analyses [8].

Secondly, we should apply new developments in causal inference methodology to overcome some failures of risk factor epidemiology [4,8]. To get causal inference from observational data right, it is wise to distinguish three classes of epidemiological tasks: (1) description, (2) prediction, and (3) causal inference [8]. Furthermore, applying a counterfactual and interventionist approach will help prevent inferential mistakes due to the confusion between a statistical association and a cause—a plague in the field of social, environmental, and life course epidemiology. This approach also matches public health needs by explicitly linking causes with interventions having an effect on the health of populations [4].

Thirdly, we should aim, systematically, for consequential epidemiology [7] and put *intervention* at the forefront of epidemiology. The concern of post-modern epidemiology should be to improve the health of populations more than tackling their determinants. We should also take distance from individual risk factor epidemiology and rather aim to solve issues at a population level, through interventions designed by data-informed and evidence-based population health science [9,10]. This will be necessary if we want to address (and not only understand) the social and environmental determinants of health [10].

In brief, for the post-modern era, I propose epidemiology to go back to the populations [11,12], and to give less weight to *explanations* and more to *descriptions* and *interventions*.

## Figures and Tables

**Table 1:** Features of traditional, modern, and post-modern epidemiology. Adapted from Pearce 1996 [1].

	Traditional Epidemiology	Modern Epidemiology	Post-Modern Epidemiology
Motivation	Public health	Science	Population health
Level of study	Population	Individual	Multiple, social, environmental; across the life course
Paradigms	Demography and social science	Biomedicine, clinical trial and biostatistics	Public health surveillance and population health monitoring; data science and causality
Epistemological approach	Realist	Positivist	Consequentialist
Level of intervention	Population	Individual	Population and individual
